# EGFR‐rich extracellular vesicles derived from highly metastatic nasopharyngeal carcinoma cells accelerate tumour metastasis through PI3K/AKT pathway‐suppressed ROS

**DOI:** 10.1002/jev2.12003

**Published:** 2020-10-30

**Authors:** Fei Li, Xin Zhao, Rui Sun, Jinxin Ou, Junyu Huang, Nanyan Yang, Ting Xu, Jingyao Li, Xiner He, Chaoyi Li, Mo Yang, Qing Zhang

**Affiliations:** ^1^ State Key Laboratory of Biocontrol School of Life Sciences Sun Yat‐sen University Guangzhou China; ^2^ Department of Nasopharyngeal Carcinoma State Key Laboratory of Oncology in South China Collaborative Innovation Center for Cancer Medicine Sun Yat‐sen University Cancer Center Guangzhou China; ^3^ Guangdong Key Laboratory of Nasopharyngeal Carcinoma Diagnosis and Therapy Guangzhou China; ^4^ The Seventh Affiliated Hospital Sun Yat‐sen University Shenzhen China; ^5^ Lianjiang People's Hospital Lianjiang China; ^6^ Institute of Sun Yat‐sen University in Shenzhen Shenzhen China

**Keywords:** epidermal growth factor receptor, extracellular vesicles, nasopharyngeal carcinoma, reactive oxygen species

## Abstract

Nasopharyngeal carcinoma (NPC) is the most common cancer with high metastatic potential that occurs in the epithelial cells of the nasopharynx. Distant metastases are the primary cause for treatment failure and mortality of NPC patients. However, the underlying mechanism responsible for the initiation of tumour cell dissemination and tumour metastasis in NPC is not well understood. Here, we demonstrated that epidermal growth factor receptor (EGFR) was highly expressed in tumour tissues of NPC patients with distant metastases and was associated with a decrease in reactive oxygen species (ROS). We also revealed that extracellular vesicles (EVs) transfer occurred from highly to poorly metastatic NPC cells, mediating cell–cell communication and enhancing the metastatic potential of poorly metastatic NPC cells. Further experiments indicated that EVs derived from highly metastatic NPC cells induced the up‐regulation of EGFR and down‐regulation of ROS in low metastatic NPC cells. Mechanistically, EGFR‐rich EVs‐mediated EGFR overexpression down‐regulated intracellular ROS levels through the PI3K/AKT pathway, thus promoting the metastatic potential of poorly metastatic NPC cells. Strikingly, treatment with EVs secreted from highly metastatic NPC cells was significantly associated with rapid NPC progression and shorter survival in xenografted mice. These findings not only improve our understanding of EVs‐mediated NPC metastatic mechanism but also have important implications for the detection and treatment of NPC patients accompanied by aberrant EGFR‐rich EVs transmission.

## 1 INTRODUCTION

Nasopharyngeal carcinoma (NPC) is a common malignant tumour that occurs in nasopharyngeal epithelial tissues and has clear regional distribution characteristics, primarily in Southeast Asia and North Africa (Lee, Lin, & Ng, 2012). Currently, the therapeutic methods of NPC are mainly radiotherapy, or combined with chemotherapy (Li, You, Xue, Tan, & Chao, 2020). Although the effect of surgical treatment has been greatly improved, the survival rate of NPC patients is still very low due to the high incidence of locoregional metastases (Chan et al., 2015; Chang, Ko, & Hong, 2004). Therefore, exploring the mechanism inducing NPC metastasis is of great value for developing more effective therapeutic drugs and novel treatment strategies for NPC patients.

Extracellular vesicles (EVs) are cell‐derived membranous particles of sub‐micrometre sizes that play important roles in cancer biology by mediating intercellular communication through carrying proteins, nucleic acids, and lipids (Reategui et al., 2018; Tadokoro et al., 2020; Wan et al., 2018). EVs comprise many subtypes, including exosomes, microvesicles, and apoptotic bodies (Wang, Tan, & Guan, 2019). Studies have shown that EVs derived from tumour cells can influence neighbouring or remote cells and tumour microenvironments to promote angiogenesis, invasiveness, and metastasis (Haga et al., 2015; Reategui et al., 2018; Sasaki et al., 2019). Tumour‐derived EVs uptake by organ‐specific cells prepares the pre‐metastatic niche, and different protein expression patterns in tumour cell‐derived EVs often determine the organ targeting of tumour metastasis (Hoshino et al., 2015; Ji et al., 2020; Qiao et al., 2020). Recently, extensive studies have suggested that NPC‐derived EVs promote tumour progression, facilitating tumour proliferation and metastasis, impeding immune response, and modulating tumour microenvironment (Mrizak et al., 2015; Ye et al., 2016; You et al., 2018). Therefore, EVs may serve as biomarkers for the diagnosis and identification of therapeutic targets for NPC. However, considering the complexity of EVs contents, further investigation is required to reveal the specific markers of NPC‐derived EVs on the pathological mechanism of the development of high metastatic potential in NPC.

Reactive oxygen species (ROS) are produced endogenously in cells in response to external stimuli, such as chemotherapeutic drugs, ionizing radiation, and inflammatory cytokines (Li et al., 2017). Intracellular ROS levels in tumour cells are higher than that in normal cells, and many studies have shown that ROS can promote the growth, invasion, and metastasis of tumour cells (Saadati et al., 2018; Storz, 2005; Vallee, Lecarpentier, & Vallee, 2019). Alternatively, ROS can also induce severe damage to cancer cells, including apoptosis, necrosis, and inhibition of distant metastases (Huang et al., 2018; Kachynski et al., 2014; Piskounova et al., 2015). There is a delicate balance between ROS production during metabolism and ROS scavenging by the antioxidant system in different cell types (Tafur & Mills, 2008). Due to the metabolic and metastatic differences of cancer cells, both ROS‐elevating and ROS‐eliminating strategies have been explored to treat cancer (Ma, Wang, Zhao, Huang, & Wu, 2019). Therefore, ROS is an indicator of different metabolic subtypes of tumour cells, and further quantitative studies are needed to determine the impact of various ROS levels and their role in tumour metastasis.

In this study, we demonstrated that the tumour tissues of NPC patients with distant metastasis presented increased EGFR expression and decreased ROS levels. EGFR‐rich EVs transfer from highly metastatic NPC cells to poorly metastatic NPC cells up‐regulated EGFR and down‐regulated the intracellular ROS levels through EGFR/PI3K/AKT axis, accelerating the progression and metastasis of NPC. These findings suggest that EVs transfer in NPC patients plays a novel non‐redundant regulatory role in the malignant transformation of NPC and reveal the potential of EGFR‐rich EVs and ROS as novel therapeutic targets for the treatment of NPC.

## 2 MATERIALS AND METHODS

### 2.1 Patients and tissue samples

Tumor tissue samples of 40 NPC patients were obtained from the Sun Yat‐sen University Cancer Center. The clinicopathological characteristics of the patients are summarized in Table 1. The tumour tissues were collected at the tumour centre for subsequent ROS detection and immunohistochemical detection of EGFR expression. Immunohistochemistry was performed using standard procedures (Liang et al., 2016). Sample collection was approved by the Hospital's Protection of Human Subjects Committee. All patients provided informed consent prior to sample collection.

**TABLE 1:** Clinical characteristics of patients with NPC

		NPC patients with distant metastases (*n* = 16)		NPC patients without distant metastases (*n* = 24)		
Characteristics	Cases	No.	%	No.	%	*P*
Gender						
Male	32	14	87.5	18	75.0	
Female	8	2	12.5	6	25.0	0.3329
Age at diagnosis (years)						
Median		47	45			
Range		37–64	16‐75			
Tumor stage						
T1‐T2	22	3	18.2	19	79.2	
T3‐T4	18	13	81.3	5	20.8	0.0002
Node stage						
N0‐N1	28	7	43.8	21	87.5	
N2‐N3	12	9	56.2	3	12.5	0.0031
Clinical stage						
I‐II	18	0	0.0	18	75.0	
III‐IV	22	16	100.0	6	25.0	<0.0001
Treatment						
RT	23	7	43.8	16	66.7	
CRT	17	9	56.2	8	33.3	0.1509

Abbreviations: CRT, chemoradiotherapy; NPC, nasopharyngeal carcinoma; RT, radiotherapy.

### 2.2 Cell culture and transfection

Human 6–10B, S26, 5–8F and S18 NPC cells were provided by the Sun Yat‐sen University Cancer Center and were cultured in RPMI‐1640 medium supplemented with 10% fetal bovine serum (Gibco). The identities of all cell lines were confirmed by short tandem repeat (STR) profiling analysis. Lipofectamine 3000 reagent (Invitrogen) was utilized for transfection according to the manufacturer's protocol. Stable cell lines were generated by transfecting NPC cells with EGFR‐expressing lentiviruses generated with pCDH‐CMV‐EGFR‐EF1‐Puro lentiviral expression vectors with psPAX2 and pMD2.G in HEK293T cells. The sequences of short hairpin RNA (shRNA) specifically targeting EGFR were 5′‐CCGGGCCACAAAGCAGTGAATTTATCTCGAGATAAATTCACTGCTTTGTGGCTTTTTG‐3′ (shEGFR‐1) and 5′‐CCGGCCTCCAGAGGATGTTCAATAACTCGAGTTATTGAACATCCTCTGGAGTTTTTG‐3′ (shEGFR‐2).

### 2.3 Generation of EGFR knock‐out cells

EGFR knock‐out (KO) 5–8F and S18 cells were generated using the CRISPR/Cas9 system as described previously (Liu et al., 2019). Briefly, two pairs of 20‐nucleotide guide RNA (gRNA) targeting exons 2 and 3 of the EGFR were constructed into lentiCRISPR v2 plasmid. Subsequently, 3 × 10^5^ cells were seeded in 6‐well plates, and Lipofectamine 3000 reagent (Invitrogen) was utilized for transfection according to the manufacturer's protocol with 4 μg of CRISPR/Cas9 plasmids. Two days after transfection, the culture medium was changed, followed by the addition of 1 μg/ml puromycin selection for the next 3 days. Single cells were seeded into 96‐well plates and cultured for 3 weeks. Finally, single cell wells were harvested for EGFR KO testing by genomic DNA sequencing and Western blot analysis. The two pair of gRNA sequences targeting EGFR's exons 2 and 3 were 5′‐TGAGCTTGTTACTCGTGCCT‐3′ and 5′‐AGGCACGAGTAACAAGCTCA‐3′ (sgRNA‐1), and 5′‐GAGTAACAAGCTCACGCAGT‐3′ and 5′‐ACTGCGTGAGCTTGTTACTC‐3′ (sgRNA‐2), respectively.

### 2.4 RNA isolation and quantitative real‐time PCR

Total RNA was extracted from NPC cells using RNAiso Plus reagent (Takara) in accordance with the manufacturer's instructions. RNA was reverse transcribed into cDNA using the RT Reagent Kit RR047A (Takara) for real‐time PCR with a SYBR Premix ExTaq Real‐Time PCR Kit (Takara). The EGFR primers were 5′‐GGCAGGAGTCATGGGAGA‐3′ and 5′‐CGATGGACGGGATCTTAG‐3′. Quantitative PCR was performed using a StepOne Plus Sequence Detection System (Applied Biosystems). All gene expression levels were normalized to GAPDH.

### 2.5 Western blot analysis

Protein extracts were boiled in RIPA buffer (Beyotime) and separated by sodium dodecyl sulfate–polyacrylamide electrophoresis gel electrophoresis (SDS‐PAGE). The proteins were then transferred to polyvinylidene fluoride membranes (Millipore) and probed with anti‐CD9, anti‐CD63, anti‐ALIX, anti‐TSG101, anti‐EGFR, anti‐FXR1, anti‐TLR‐4, anti‐MMP2, anti‐TGF‐β1, anti‐phospho‐STAT5, anti‐STAT5, anti‐phospho‐ERK1/2, anti‐ERK1/2, anti‐phospho‐AKT, anti‐AKT, anti‐Ki‐67, anti‐PTEN (Abcam), anti‐Bcl‐2, anti‐Bax, anti‐E‐Cadherin, anti‐Vimentin, and anti‐GAPDH antibodies (Cell Signaling Technology). Electrochemiluminescence (Millipore) was applied to determine protein expression levels.

### 2.6 EVs isolation, identification, and internalization

For the co‐culture system, mCherry‐CD63‐labeled 5–8F and S18 cells were inoculated in the upper chamber. 6–10B and S26 were inoculated in the lower chamber. After 48 h of culture, the cells in the lower chamber were stained with Hoechst 33342 (Sigma) and observed using LSM880 laser confocal microscope (Zeiss). For EVs isolation, NPC cells were cultured in EVs‐depleted mediums for 48 h. Subsequently, 10 ml of culture media was used for EVs collection as described previously (Chan et al., 2015). Briefly, the culture medium was subjected to three rounds of 30‐min centrifugation at increasing accelerations (200 × *g*, 1200 × *g*, and 20,000 × *g*) to remove cells and cellular debris. The remaining solution was filtered through a 0.22‐μm membrane. EVs were pelleted at 120,000 × *g* for 2 h at 4°C (SW 41Ti, Beckman). The pellet was washed in PBS solution followed by a second spin at 120,000 × *g* for 2 h at 4°C (SW 60Ti, Beckman) and resuspended in 100 μl of PBS solution. For EGFR‐KO EVs isolation, EGFR‐KO 5–8F and S18 cells were cultured in EVs‐depleted mediums for 48 h and EGFR‐KO EVs were isolated as described above. To ensure the removal of EGFR‐positive EVs, EVs isolated from EGFR‐KO 5–8F and S18 cells were incubated for 2 h with EGFR antibody and precipitated with protein G‐Sepharose to remove EGFR‐positive EVs. All isolated EVs were characterized according to the MISEV 2018 guidelines (Thery et al., 2018). The protein quantity of EVs was determined using the BCA protein assay kit (Thermo Scientific). We have submitted all relevant data of our experiments to the EV‐TRACK knowledgebase (EV‐TRACK ID: EV200091) (Van Deun et al., 2017). Purified EVs were labelled with the red fluorescent linker PKH26 (Sigma) according to the manufacturer's instructions, followed by co‐culturing with 6–10B and S26 cells for 2 h. The cells were then washed with PBS solution and fixed in 4% paraformaldehyde. The membrane and nuclei of the cells were stained by Dio (Solarbio) and Hoechst 33342, respectively, and imaged by confocal microscope as mentioned above.

### 2.7 Intracellular ROS detection

The intracellular ROS generation was detected using a ROS assay kit (Abcam) according to the manufacturer's instructions. Briefly, adherent NPC cells, tumour single cell suspensions, or tumour tissue sections of NPC patients were incubated with DCFH‐DA probe at 1:1000 dilution in serum‐free RPMI medium at 37°C for 30 min, then washed with serum‐free RPMI medium three times and visualized under a LSM880 laser confocal microscope (Zeiss) or detected by a fluorescent reader (TECAN) with excitation at 488 nm.

### 2.8 Nanoparticle tracking analysis

The size distribution and concentration of EVs isolated from NPC cells were analysed using NanoSight LM10 (NanoSight). EVs suspensions were diluted between 1:50 to 1:500 in PBS solution to achieve a concentration range of 10^7^–10^8^ nanoparticles per ml. Data were obtained as the mean reading of three 1‐min videos with parameters being set at a camera level of 12 and detection threshold of 3 as described previously (Duong, Chung, Bouchareychas, & Raffai, 2019). Captured video was analysed using NTA software (version 3.2 Build 16), and the average size distribution graph was plotted using GraphPad Prism 5.

### 2.9 Clone formation assay

NPC cells treated for 24 h with 5 μg/ml of EVs derived from highly metastatic NPC cells (H‐EVs) or 5 μg/ml of EVs derived from low metastatic potential NPC cells (L‐EVs) were transferred into 24‐well plates at 400 cells/well. To each well, 500 μl complete medium was added, and cells were cultured until cell clones could be observed directly. After the medium was removed, clones were fixed in 4% paraformaldehyde for 20 min and stained with 0.1% crystal violet for counting.

### 2.10 Wound healing assay

Culture‐Insert (Ibidi) was used to perform wound healing assay to measure cell migration according to the manufacturer's instructions. Briefly, NPC cells were seeded onto 24‐well plates to create a confluent monolayer and Culture‐Insert was inserted to form a “scratch” simultaneously. After culturing for 12 h, Culture‐Insert was removed to form scratches and the first image of the scratch was acquired with a 20 × objective using a Nikon Eclipse Ti2‐E microscope (Nikon). The second image was acquired after H‐EVs (5 μg/ml) or L‐EVs (5 μg/ml) treatment for 24 h.

### 2.11 Invasion assay

A total of 1 × 10^5^ NPC cells were seeded into the Matrigel (200 μg/ml)‐coated upper chamber of a polycarbonate transwell filter chamber (Corning) and separately co‐incubated with H‐EVs (5 μg/ml) or L‐EVs (5 μg/ml) for 24 h. After non‐invading cells were removed from the top chamber with a cotton swab, cells on the lower membrane surface were fixed in 4% paraformaldehyde, stained with 0.1% crystal violet, and photographed in three independent 20 × fields for each well.

### 2.12 Immunofluorescence assay

6–10B and S26 cells were grown on glass coverslips and treated with or without H‐EVs (5 μg/ml) for 48 h. After being fixed in 4% paraformaldehyde and permeabilized in 0.1% Triton X‐100, cells were blocked in 5% BSA‐PBS solution, and incubated with primary anti‐human E‐cadherin antibodies and primary anti‐human vimentin antibodies or primary anti‐human EGFR antibodies. FITC‐conjugated and Alexa Fluor 555‐conjugated IgG secondary antibodies were used for E‐cadherin and vimentin/EGFR labelling, respectively. Cell nuclei were stained with Hoechst 33342 and imaged with a 63 × objective using an LSM 880 confocal microscope (Zeiss).

### 2.13 Total RNA isolation and expression analysis

6–10B and S26 cells were treated with or without H‐EVs (5 μg/ml) for 48 h. Total RNA was extracted using TRIzol (Invitrogen) according to the manufacturer's instructions. cDNA libraries were constructed following the High‐Throughput Illumina Strand‐Specific RNA Sequencing Library protocol. The library products were sequenced on an Illumina HiSeq 2500. RNA‐library sequencing and transcript assembly were performed by Biomarker Technologies, Inc. All usable reads that could be uniquely mapped to a gene were used to calculate the expression level. The resulting gene expression profiles were measured by assessing the number of gene reads as previously described (Mortazavi, Williams, McCue, Schaeffer, & Wold, 2008). Different samples were used to calculate logarithmic fold change in the expression levels of selected differential genes using the following criteria: false discovery rate ≤0.001 and a log 2‐fold change ratio ≥1. All raw data have been deposited under the Gene Expression Omnibus accession number GSE149714.

### 2.14 Animal models

Five‐week‐old BALB/c nude mice were maintained in the Laboratory Animal Center of Sun Yat‐sen University and procedures were performed according to the institutional ethical guidelines for animal experiments. NPC xenograft mice were generated by co‐injecting 1 × 10^6^ 6–10B or S26 cells with or without H‐EVs (10 μg) into the abdominal cavity of BALB/c nude mice. After 20 days, tumour cells from ascites were collected and selected with puromycin for Western blot and ROS detection, and alternatively used for subcutaneous injection to obtain subcutaneously xenografted mice. After 2 weeks of *in situ* H‐EVs (10 μg) injections, transplanted mice were used for Histological and Kaplan‐Meier survival analysis.

### 2.15 Histological analysis

Lung and liver tissues collected from NPC subcutaneously transplanted mice treated with or without H‐EVs (10 μg) were fixed with 4% polyphosphate formaldehyde, embedded in paraffin, and cut into 5‐μm sections. Histopathological changes were assessed using standard haematoxylin and eosin (H&E) staining.

### 2.16 Statistical analysis

All data were reported as the mean ± SD of three independent experiments. Statistical analysis was performed using the paired or unpaired two‐tailed Student's t‐test or one‐way analysis of variance. For mouse Kaplan‐Meier survival curves, P‐values were produced using a log‐rank (MantelCox) test. *^:^P* < 0.05, *^::^P* < 0.01, and *^:::^P* < 0.001 were considered statistically significant.

## 3 RESULTS

### 3.1 Low ROS levels are correlated with increased EGFR expression in both highly metastatic NPC cells and tumour tissues of NPC patients

**FIGURE 1:**
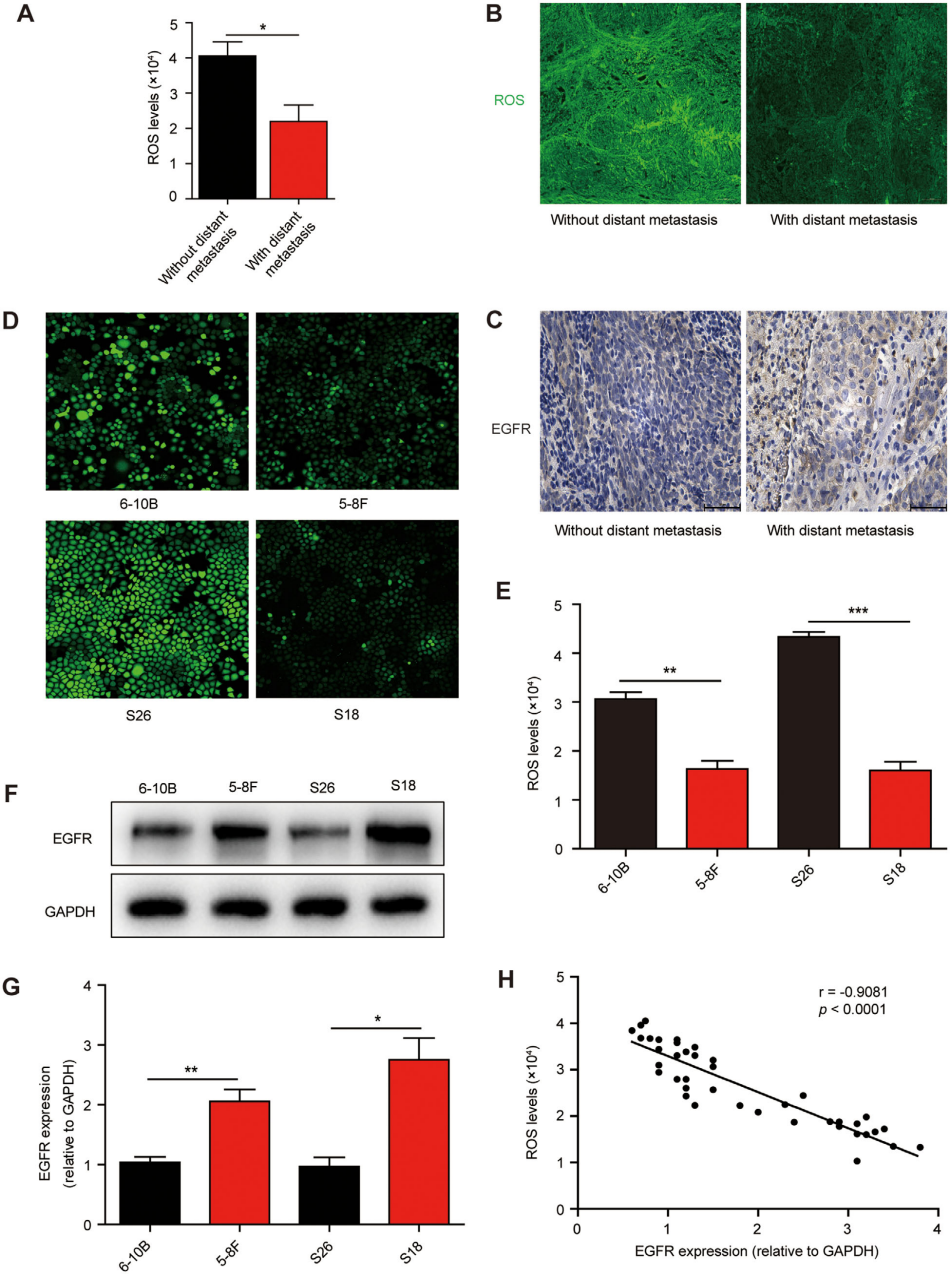
ROS is reduced in NPC cells and patients with high metastatic potential and is correlated with an increase in EGFR. (a, b) Tumour single cell suspensions and tumour tissue sections of NPC patients with effective distant metastases (*n* = 16) and without distant metastases (*n* = 24) were incubated with DCFH‐DA probe and *in situ* intracellular ROS levels were analysed using (a) fluorescence microplate reader or (b) confocal microscope (Magnification × 200; Scale bar, 36.67 μm). (c) Immunohistochemical staining showing EGFR expression in tumour tissue of NPC patients with effective distant metastases (*n* = 16) and without distant metastases (*n* = 24) (Magnification × 400; Scale bar, 50 μm). (d) Intracellular ROS production of NPC cells 6–10B, 5–8F, S26 and S18, assessed using DCFH‐DA probe and recorded using confocal laser scanning microscope. (e) Levels of intracellular ROS production in NPC cells, quantified by DCFH‐DA probe and analysed using fluorescence microplate reader. (f) Western blot analysis of EGFR expression in NPC cells. GAPDH was used a loading control. (g) Quantitative PCR analysis of EGFR expression in NPC cells. Values were normalized to GAPDH. (h) A nonlinear correlation between intratumoral ROS levels and EGFR expression. The EGFR expression in tumour tissues of NPC patients (*n* = 40) was normalized to GAPDH. Experiments were performed in triplicate. Data are presented as mean ± SD (*^:^P* < 0.05; *^::^P* < 0.01; *^:::^P* < 0.001)

Considering the uncertainty of the role of ROS in regulating metastasis for different tumour cells, we collected tumour tissues of NPC patients with or without distant metastases to assess intratumoral ROS levels. Unsurprisingly, we found that efficiently metastasizing NPC tissues had lower ROS levels compared with poorly metastasizing NPC tissues (Figure 1a and Figure 1b), indicating that the high ROS levels may inhibit the metastasis of NPC cells. Meanwhile, these efficiently metastasizing NPC tissues were associated with increased EGFR expression (Figure 1c). Further observation of ROS in living NPC cells by fluorescence microscopy revealed lower ROS levels in highly metastatic human NPC cells (5‐8F and S18) compared with that of poorly metastatic NPC cells (6‐10B and S26) (Figure 1d). Similar results were obtained by fluorescently detecting ROS (Figure 1e). Notably, we found that the expression of EGFR in 5–8F and S18 was more than twice as high as that of EGFR in 6–10B and S26, which was further confirmed by quantitative real‐time PCR (Figure 1f and Figure 1g). Taken together, these data demonstrate that decreased intratumoral ROS levels are correlated with increased EGFR expression in metastasizing NPC tissues (Figure 1h) and, therefore, ROS and EGFR may play a novel non‐redundant regulatory role in NPC metastasis.

### 3.2 EVs transfer from highly metastatic NPC cells to poorly metastatic NPC cells mediates cell‐cell communication

**FIGURE 2:**
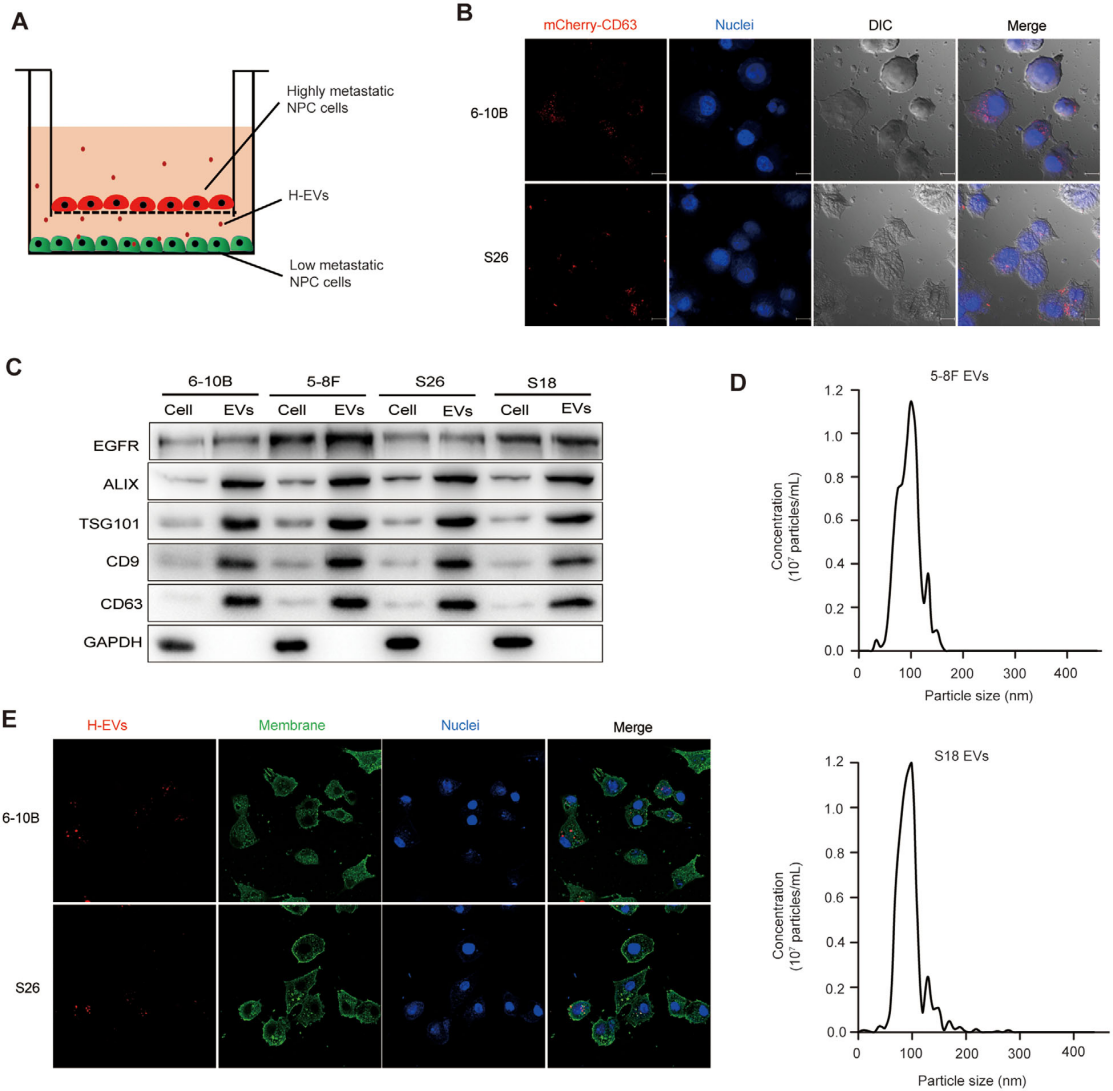
EVs transfer from highly metastatic NPC cells to poorly metastatic NPC cells mediates cell‐cell communication. (a) Schematic representation of highly and poorly metastatic NPC cells co‐culture system. pmCherry‐CD63‐transfected highly metastatic NPC cells were seeded into the upper chamber and poorly metastatic NPC cells were seeded into the lower chamber. (b) Confocal microscopy observation of the internalization of EVs secreted from mCherry‐CD63‐labeled 5–8F or S18 (upper chamber) in 6–10B or S26 cells (lower chamber). Hoechst 33342 was used to stain cellular nuclei (blue fluorescence). Stereoscopic structure of cells was observed by differential interference contrast (DIC) (Magnification × 630; Scale bar, 10 μm). (c) Western blot analysis showing the presence of CD9, CD63, ALIX, TSG101, and EGFR and absence of GAPDH in EVs derived from the conditioned medium of NPC cells. (d) Representative nanoparticle tracking analysis plots showing the size distribution and total number of EVs isolated from the same volume of conditioned medium of 5–8F and S18 cells. (e) Confocal microscopy image showing the internalization of PKH26‐labeled EVs (red) isolated from the conditioned medium of highly metastatic NPC cells by 6–10B and S26 cells. Hoechst 33342 was used to stain the nuclei of cells (blue). Cell membrane was stained by Dio (green fluorescence). (Magnification × 630; Scale bar, 10 μm). Experiments were performed in triplicate

Although ROS and EGFR express at different levels between highly and poorly metastatic NPC cells, it remains unclear whether there is a direct way to communicate between highly and poorly metastatic NPC cells. Studies have shown that EVs, as tiny vesicles secreted by cells, can deliver lipids, proteins and nucleic acids between cells and mediate intercellular communication (Colombo, Raposo, & Thery, 2014; Fujita, Kosaka, Araya, Kuwano, & Ochiya, 2015; Kowal et al., 2016). Therefore, we designed a co‐culture system of high and low metastatic potential NPC cells in which the highly metastatic NPC cells (upper chamber) were transfected with pMcherry‐CD63 plasmid to label the EVs marker CD63 (Figure 2a). After 24 h of co‐culture, the poorly metastatic NPC cells (lower chamber) were observed by laser confocal microscopy, and it was found that a large number of CD63‐labelled vesicles were internalized into these low metastatic potential NPC cells (Figure 2b). This suggested that the EVs secreted by highly metastatic NPC cells may be absorbed by poorly metastatic NPC cells for intercellular communication. Subsequently, we isolated EVs from cultured NPC cells supernatants and characterized EVs isolates by detecting enrichment of membrane (CD9 and CD63) and non‐membrane (ALIX and TSG101) EVs markers (Figure 2c). The size distribution of highly metastatic NPC cell (5‐8F and S18)‐derived EVs (H‐EVs) was determined specifically using Nanoparticle Tracer Analysis. The majority of the EVs from both cell lines were in the accepted size range in which the peaks for particle size of 5–8F EVs were 80–120 nm, and that of S18 EVs were 80–150 nm (Figure 2d). For further confirmation of the uptake of EVs from highly metastatic NPC cells by low metastatic NPC cells, H‐EVs were labelled with fluorescent PKH26 and co‐cultured with 6–10B or S26 cells. The PKH26‐labelled H‐EVs were internalized by 6–10B and S26 cells during a 2‐h incubation as measured by fluorescence microscopy (Figure 2e). Our results indicate that EVs secreted by NPC cells mediate the communication of information between highly and poorly metastatic NPC cells, and therefore affect the metastatic process of NPC cells.

### 3.3 Uptake of highly metastatic NPC cell‐derived EVs promotes metastasis of NPC cells

**FIGURE 3:**
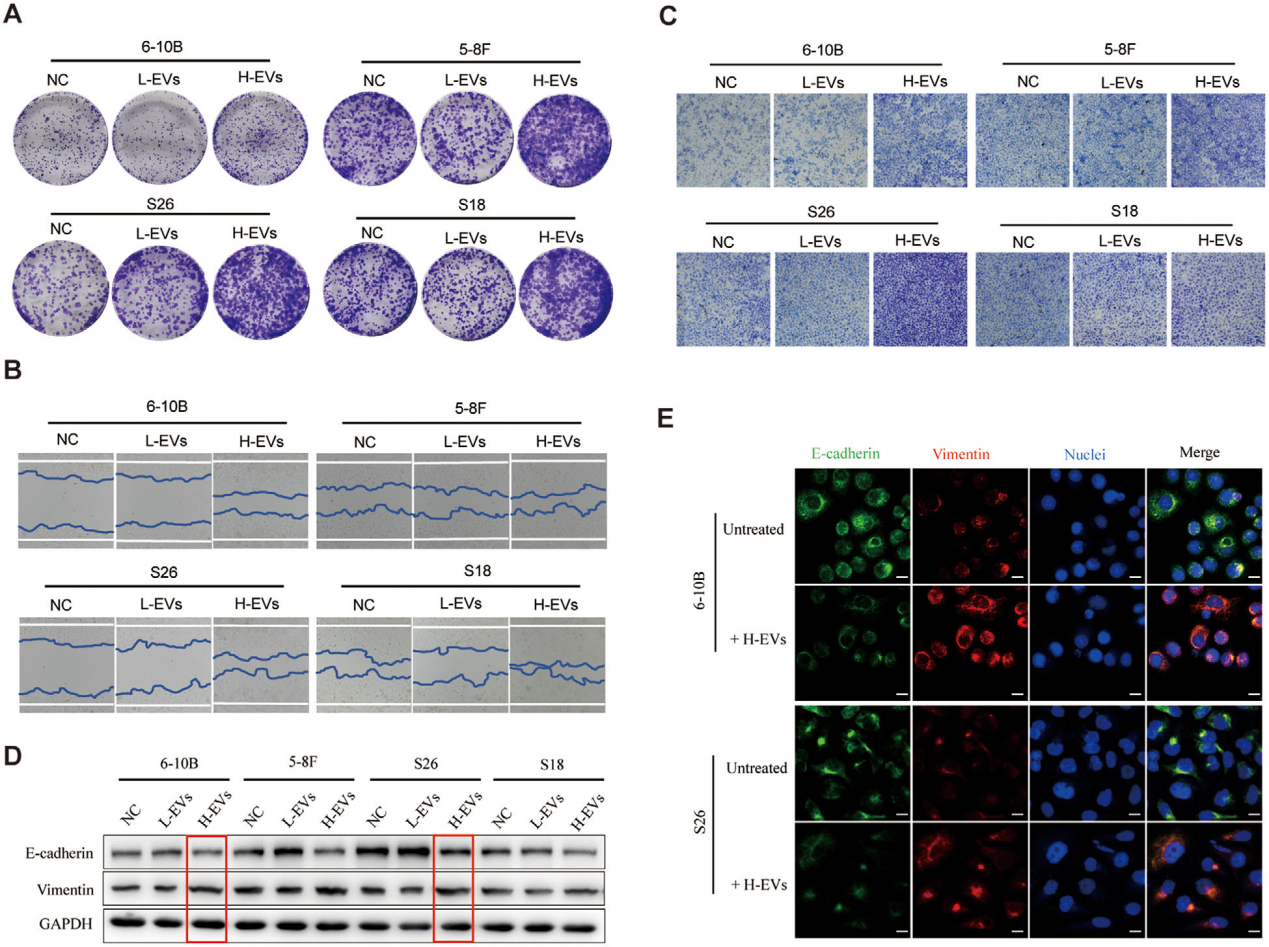
Uptake of EVs derived from highly metastatic NPC cells enhances NPC cells metastasis. (a) The effect of L‐EVs and H‐EVs on NPC cells clone formation. (b) Wound healing assay showing effects of L‐EVs and H‐EVs internalization on NPC cells migration. (c) Effect of L‐EVs and H‐EVs internalization on NPC cells invasion by Matrigel invasion assay. (d) Western blot for EMT markers E‐cadherin and Vimentin after treatment with L‐EVs and H‐EVs for 48 h. (e) Expression of proteins involved in EMT was confirmed by immunofluorescence after treatment with H‐EVs for 48 h. Stained cells were examined with E‐cadherin‐ and Vimentin‐specific antibodies and protein localization was recognized by FITC (green) and Alexa Fluor 555 (red) secondary antibodies. Hoechst 33342 was used to stain the nuclei of cells (blue) (Magnification × 630; Scale bar, 10 μm). Experiments were performed in triplicate

Considering that H‐EVs can be internalized by poorly metastatic NPC cells to mediate intercellular communication, we speculated that there would be a series of changes in the metastatic capacity of poorly metastatic NPC cells after absorbing these EVs. Therefore, the clonogenic ability of NPC cells was assessed. We demonstrated that following treatment with H‐EVs, cellular clonogenic ability was enhanced compared with the untreated group. Conversely, there was a slight, though not statistically significant, suppressed effect of low metastatic NPC cell‐derived EVs (L‐EVs) treatment on the clone‐forming ability of NPC cells (Figure 3a). Wound healing assays also showed that H‐EVs treatment significantly increased the migration of NPC cells, compared with vehicle and L‐EVs treatment (Figure 3b). In accordance with the migration results, cell invasion was significantly increased in NPC cells by H‐EVs treatment, compared with vehicle and L‐EVs treatment (Figure 3c), indicating that the uptake of H‐EVs promotes the metastasis of NPC cells.

Further analysis of the EVs communication between NPC cells was conducted by looking at the signalling pathways associated with metastasis. The epithelial‐mesenchymal transition (EMT) is a key pathological transformation procedure that drives cancer metastasis through the conspicuous down‐regulation of epithelial markers and loss of intercellular junctions, resulting in a loss of epithelial polarity and reduced intercellular adhesion (Okada et al., 2015; Xie et al., 2017). To explore whether EMT was induced by H‐EVs or L‐EVs in NPC cell lines, we examined EMT molecular markers, E‐cadherin and vimentin, in NPC cells during treatment with H‐EVs or L‐EVs. Interestingly, we found that H‐EVs treatment caused a reduction of E‐cadherin and increase of vimentin in 5–8F and S18 (Figure 3d). Furthermore, 6–10B and S26 cells treated with H‐EVs, but not L‐EVs, also demonstrated a loss of E‐cadherin expression and gain of vimentin expression (Figure 3d). Subsequently, double immunofluorescence staining further confirmed that treatment with H‐EVs led to a decrease of E‐cadherin and increase of vimentin in 6–10B and S26 cells (Figure 3e). Overall, these results demonstrate that the uptake of H‐EVs promote the metastasis of low metastatic NPC cells through the EMT process.

### 3.4 H‐EVs induce EGFR up‐regulation and ROS down‐regulation in low metastatic NPC cells

**FIGURE 4:**
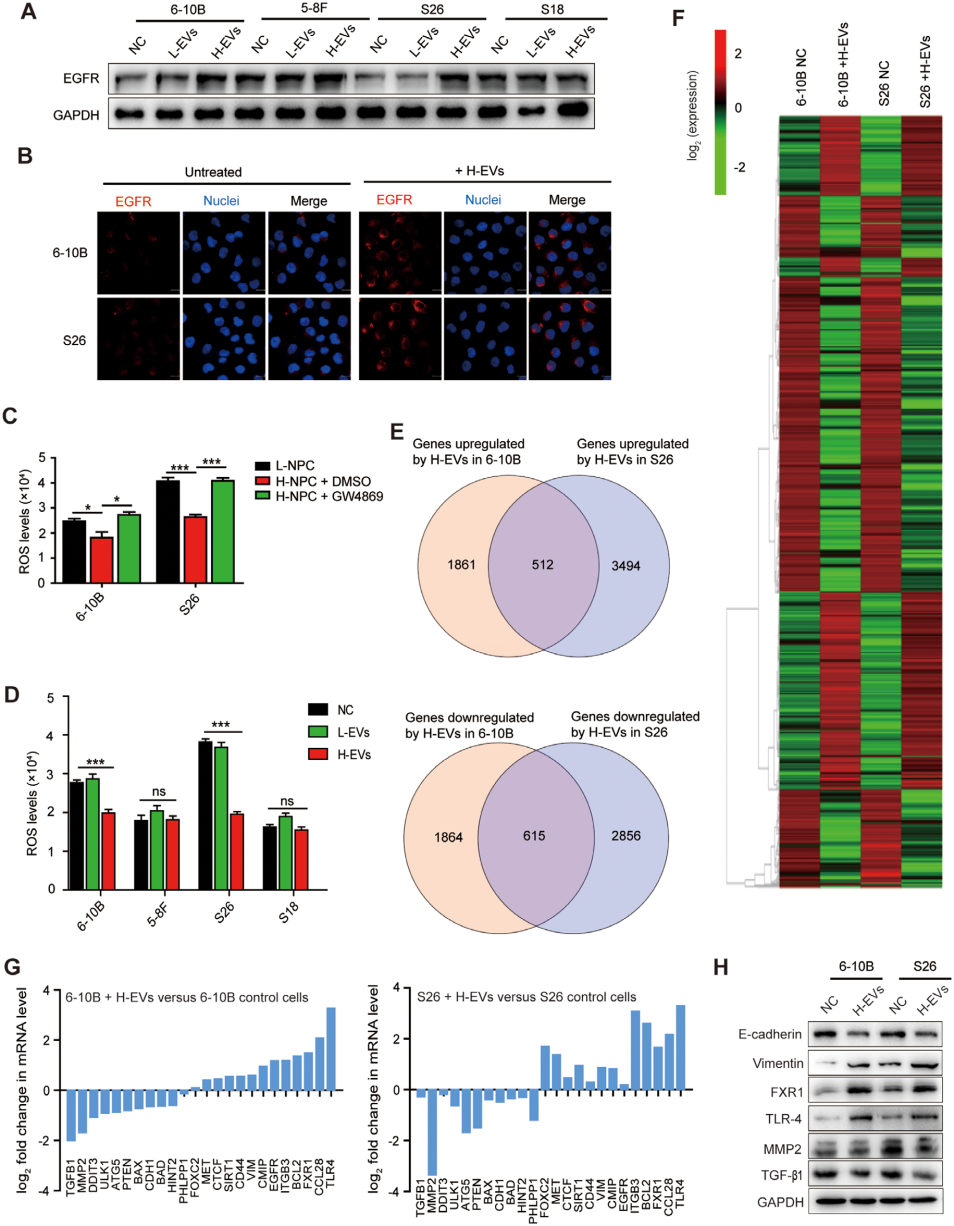
H‐EVs induces EGFR up‐regulation and ROS down‐regulation in low metastatic NPC cells. (a) Western blot analysis of EGFR expression in NPC cells following treatment with L‐EVs and H‐EVs. (b) EGFR expression by confocal microscope in 6–10B and S26 cells pretreated with H‐EVs. Stained cells were examined with EGFR‐specific antibodies, and protein expression was recognized by Alexa Fluor 555 (red) secondary antibodies. Hoechst 33342 was used to stain the nuclei of cells (blue). (Magnification × 630, Scale bar, 10 μm). (c) Detection of intracellular ROS in 6–10B and S26 cells (lower chamber) in co‐culture system following treatment with or without GW4869 in highly metastatic NPC cells (upper chamber). (d) Detection of intracellular ROS in NPC cells following treatment with L‐EVs or H‐EVs. (e) Expression of potential genes altered by H‐EVs treatment; numbers indicate quantity of genes in each DEG subset. (f) Heatmap displaying hierarchical clustering of genes in 6–10B and S26 cells in response to H‐EVs treatment. Gene expression values are displayed by applying progressively brighter shades of red (up‐regulated) or green (down‐regulated). (g) Column diagram represents the expression of genes correlated with tumor metastasis with H‐EVs treatment in 6–10B and S26 cells. (h) Western blot analysis for validation of the identified genes correlated with tumor metastasis after H‐EVs treatment in 6–10B and S26 cells. Experiments were performed in triplicate. Data represent the mean ± SD (*^:^P* < 0.05; *^::^P* < 0.01; *^:::^P* < 0.001)

Since the EGFR signalling pathway is closely related to tumour cell growth, invasion, angiogenesis and metastasis (Hynes & Lane, 2005; Lee et al., 2017), we detected the expression of EGFR in NPC cells and NPC cell‐derived EVs, and found that EGFR was enriched in highly metastatic NPC cells and H‐EVs compared with low metastatic NPC cells and L‐EVs (Figure 2c). To investigate the detailed mechanism by which EGFR‐enriched H‐EVs internalization promotes low metastatic NPC cells metastasis, we co‐cultured H‐EVs with NPC cells, and found that H‐EVs internalization up‐regulated the expression of EGFR in 6–10B and S26 cells, reaching the expression levels of EGFR in 5–8F and S18, respectively (Figure 4a). Subsequent immunofluorescence studies also confirmed that H‐EVs treatment up‐regulated the expression of EGFR in 6–10B and S26 cells (Figure 4b), suggesting that H‐EVs may promote the metastasis of poorly metastatic NPC cells by increasing the expression of EGFR to a level similar to that in highly metastatic NPC cells. Notably, in our co‐culture system of highly and poorly metastatic NPC cells (Figure 2a), the intracellular ROS levels of the low metastatic NPC cells (lower chamber) co‐cultured with the highly metastatic NPC cells decreased significantly, but treatment with GW4869, an EVs inhibitor (Umezu et al., 2019), repaired the reduction in ROS levels (Figure 4c). Low metastatic NPC cells were subsequently co‐cultured with H‐EVs, followed by detection of intracellular ROS. This result further corroborated that H‐EVs treatment suppressed ROS production in low metastatic NPC cells (Figure 4d). Moreover, comparative transcriptome analyses of differentially expressed genes revealed that there was a significant overlap in the expression of ROS regulatory genes that were altered in 6–10B and S26 upon H‐EVs treatment (Figure 4e). EGFR and the majority of ROS production‐suppressing genes, including FXR and TLR‐4 (Fang et al., 2018; Wang et al., 2015), were significantly increased in H‐EVs treated 6–10B and S26 cells. In contrast, the expression levels of ROS production‐promoting genes (such as MMP2 and TGF‐β1) (Barhoumi et al., 2017; Fan et al., 2019) were significantly lower in H‐EVs‐treated 6–10B and S26 cells than untreated 6–10B and S26 cells (Figure 4f and Figure 4g), which was further confirmed by Western blot analysis (Figure 4h). Collectively, these data indicate that H‐EVs‐induced EGFR up‐regulation is concomitant with decreased ROS production in low metastatic NPC cells and may therefore play a novel metastasis‐promoting effect in NPC cells.

### 3.5 H‐EVs‐mediated EGFR overexpression down‐regulates ROS via PI3K/AKT pathway to promote NPC metastasis

**FIGURE 5:**
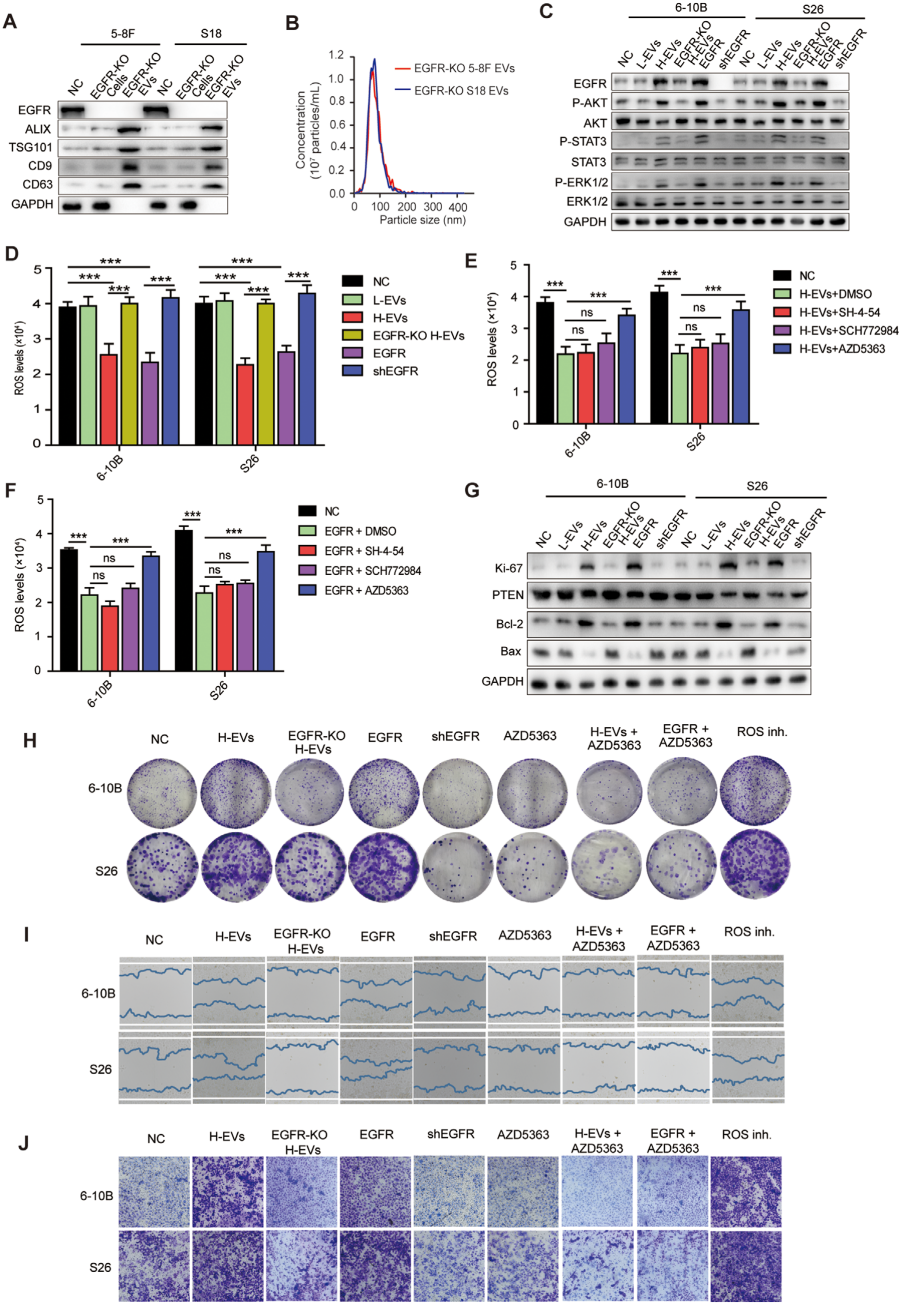
H‐EVs‐induced EGFR expression down‐regulates ROS via the PI3K/AKT pathway to promote metastasis of NPC cells. (a) Western blot analysis showing the presence of CD9, CD63, ALIX, and TSG101 and absence of GAPDH in EGFR‐KO EVs derived from the conditioned medium of EGFR‐KO 5–8F and S18 cells. (b) Representative NTA plots showing the size distribution and total number of EVs isolated from the same volume of conditioned medium of EGFR‐KO 5–8F and S18 cells. (c) Western blot analysis of phospho‐STAT3, STAT3, phospho‐AKT, AKT, phospho‐ERK1/2, and ERK1/2 in EVs‐treated, EGFR‐KO H‐EVs‐treated, EGFR overexpressed, and shEGFR transfected 6–10B and S26 cells. (d) Intracellular ROS levels in L‐EVs‐, H‐EVs‐ or EGFR‐KO H‐EVs‐treated, EGFR overexpressed, and shEGFR transfected 6–10B and S26 cells by fluorescence microplate reader. (e) Intracellular ROS levels in H‐EVs‐treated 6–10B and S26 cells pretreated with DMSO control, SH‐4‐54, SCH772984, or AZD5363. (f) Intracellular ROS levels in EGFR overexpressed 6–10B and S26 cells pretreated with DMSO control, SH‐4‐54, SCH772984, or AZD5363. (g) Western blot analysis of Ki‐67, PTEN, Bcl‐2, and Bax in L‐EVs‐, H‐EVs‐, or EGFR‐KO H‐EVs‐treated, EGFR overexpressed, and shEGFR 6–10B and S26 cells. (h) Clone formation capacity of H‐EVs‐treated, EGFR‐KO H‐EVs‐treated, EGFR overexpressed, ROS inhibited, shEGFR, AZD5363 treated, H‐EVs and AZD5363 co‐treated, and EGFR overexpression combined with AZD5363 treated 6–10B and S26 cells, respectively. (i) Wound healing assay showing cell migration of H‐EVs‐treated, EGFR‐KO H‐EVs‐treated, EGFR overexpressed, ROS inhibited, shEGFR,AZD5363 treated, H‐EVs and AZD5363 co‐treated, and EGFR overexpression combined with AZD5363 treated 6–10B and S26 cells. (j) Cell invasion of H‐EVs‐treated, EGFR‐KO H‐EVs‐treated, EGFR overexpressed, ROS inhibited, shEGFR, AZD5363 treated, H‐EVs and AZD5363 co‐treated, and EGFR overexpression combined with AZD5363 treated 6–10B and S26 cells using a Matrigel invasion assay. Experiments were performed in triplicate. Data are presented as mean ± SD (*^:^P* < 0.05; *^::^P* < 0.01; *^:::^P* < 0.001)

Many studies have shown that the JAK/STATs, MAPK/ERK and PI3K/AKT pathways are aberrantly activated in EGFR‐expressed tumour cells, which is thought to be responsible for cancer cell growth and metastasis (Tang et al., 2019; van der Mijn et al., 2014; Yang, Qian, Li, Li, & Han, 2018; Zhao & Hu, 2013). We have demonstrated that EGFR expression was up‐regulated in low metastatic NPC cells after absorbing H‐EVs to further facilitate low metastatic NPC cells invasion and metastasis. To investigate the detailed mechanism by which H‐EVs‐induced EGFR overexpression promotes NPC cells invasion and metastasis, we used CRISPR/Cas9 technology to generate EGFR‐KO 5–8F and S18 cells. The EGFR‐KO H‐EVs were then isolated from these EGFR‐KO highly metastatic NPC cells (Figure 5a and Figure 5b). Additionally, we designed two lentivirus‐encoding shRNA targeting two distinct sequences of EGFR and generated stable EGFR knockdown 6–10B and S26 cell lines. When treated with H‐EVs or overexpressed EGFR, abnormal activation of AKT, STAT3 and ERK1/2 signalling was found in 6–10B and S26 cells, but this activation was abolished by EGFR KO in H‐EVs or EGFR knockdown in 6–10B and S26 cells (Figure 5c). In parallel with this, intracellular ROS detection showed that both H‐EVs treatment and EGFR overexpression could induce the decrease of ROS levels in 6–10B and S26 cells, which was reversed in these cells with EGFR‐KO H‐EVs treatment or EGFR knockdown (Figure 5d), implying that H‐EVs induced up‐regulation of EGFR reduces ROS levels in NPC cells. Subsequently, we applied small‐molecular inhibitors of STATs (SH‐4‐54), ERK1/2 (SCH772984), and AKT (AZD5363) to suppress H‐EVs‐ and EGFR‐mediated signalling pathways in 6–10B and S26 cells. Unexpectedly, although H‐EVs treatment and EGFR overexpression decreased the intracellular ROS levels of 6–10B and S26, AZD5363 treatment, rather than SH‐4‐54 and SCH772984, abolished the H‐EVs‐ and EGFR‐induced ROS reduction in 6–10B and S26 cells (Figure 5e and Figure 5f), indicating that EGFR signalling primarily regulates intracellular ROS via the PI3K/AKT pathway.

In addition, treatment with H‐EVs and overexpressed EGFR induced up‐regulation of the proliferation marker Ki‐67, down‐regulation of the tumor‐suppressor gene PTEN, and activation of anti‐apoptotic responses (increased Bcl‐2 and inhibited Bax expression) in 6–10B and S26 cells, which was inverted by EGFR‐KO H‐EVs treatment or EGFR knockdown (Figure 5g). Similarly, RNA‐Seq analyses of differentially expressed genes revealed that the majority of the genes correlated with anti‐apoptotic responses and pro‐metastasis could be potentially regulated by treating 6–10B and S26 cells with H‐EVs, including Bcl‐2 and CD44, which were significantly increased in H‐EVs‐treated 6–10B and S26 cells (Figure 4f and Figure 4g).

As we have shown, H‐EVs uptake can promote the metastasis of low metastatic NPC cells and H‐EVs‐mediated EGFR up‐regulation can reduce the intracellular ROS level of low metastatic NPC cells through the PI3K/AKT pathway, we therefore sought to investigate the association between tumour metastasis and EGFR‐induced ROS reduction. Clone formation assays demonstrated that H‐EVs treatment, EGFR overexpression, and ROS inhibition, respectively, dramatically promoted the abilities of colony formation in 6–10B and S26 cells. However, EGFR KO in H‐EVs or combined AZD5363 treatment led to statistically significant inhibition of H‐EVs‐ and EGFR‐induced colony formation in 6–10B and S26 cells (Figure 5h). Subsequent wound healing assays and invasion assays also highlighted that H‐EVs treatment, EGFR overexpression, and ROS inhibition enhanced cell migration and invasion in 6–10B and S26 cells, respectively. However, the promotion of migration and invasion in both cell lines were effectively reversed by EGFR KO and AZD5363 treatment (Figure 5i and Figure 5j). Taken together, these results indicate that H‐EVs uptake upregulates the expression of EGFR in low metastatic NPC cells, and then inhibits intracellular ROS levels to promote low metastatic NPC cells metastasis through the PI3K/AKT pathway.

### 3.6 H‐EVs‐induced EGFR up‐regulation and ROS reduction enhance low metastatic NPC cell growth and metastasis in a xenograft model

**FIGURE 6:**
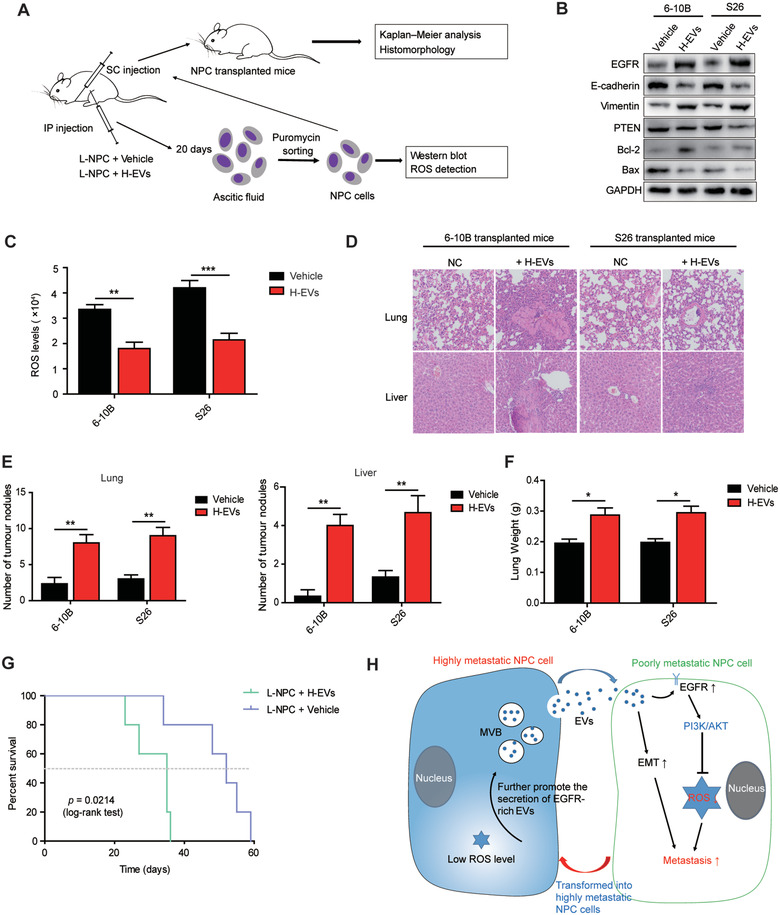
H‐EVs treatment promotes the metastasis of NPC cells and causes shortened survival of NPC‐bearing mice. (a) Experimental design of cell transplantation with 6–10B and S26 cells by intraperitoneal injection (IP) and subcutaneous (SC) injection, respectively, and subsequent *in vivo* studies and NPC‐ascites sorting and monitoring. (b) Western blot analysis of EGFR, E‐cadherin, Vimentin, PTEN, Bcl‐2, and Bax in 6–10B and S26 ascitic cells developed from intraperitoneally injected cells following treatment with or without H‐EVs. (c) Intracellular ROS detection of 6–10B and S26 ascitic cells following intraperitoneally co‐injected H‐EVs. (d) Histological analysis of the lung and liver tissues obtained from 6–10B‐ and S26‐transplanted mice following treatment with or without H‐EVs (Magnification × 200; Scale bar, 10 μm). (e) Number of tumor nodules in the lung and liver metastases of 6–10B and S26 following treatment with or without H‐EVs. (f) Wet lung weight of 6–10B‐ and S26‐transplanted mice following treatment with or without H‐EVs. (g) Kaplan‐Meier survival curves for mice subcutaneously transplanted with 6–10B and S26 cells following treatment with or without H‐EVs. P values were determined by the log‐rank (Mantel‐Cox) test. (h) Schematic illustrating the transit of highly metastatic NPC cell‐derived EVs to poorly metastatic NPC cells to drive low metastatic potential cells toward a highly metastatic phenotype. All experiments were performed in triplicate. Data are presented as mean ± SD (*^:^P* < 0.05; *^::^P* < 0.01; *^:::^P* < 0.001)

To further elucidate the role of H‐EVs in low metastatic NPC cell growth and metastasis, 6–10B and S26 (1 × 10^6^) cells carrying puromycin resistance were injected intraperitoneally in BALB/c nude mice followed by injection with H‐EVs (10 μg) to generate an ascitic tumour mouse model. Ascitic cells were subsequently selected using puromycin resistance for *in vitro* detection and a subcutaneous transplantation mouse model (Figure 6a). Consistent with *in vitro* results, H‐EVs treatment increased EGFR expression and activated an EMT program in ascitic cells derived from 6–10B and S26, which was accompanied by elevated levels of the anti‐apoptotic response and suppressed expression of PTEN (Figure 6b). Meanwhile, ROS detection also confirmed that H‐EVs‐treated ascitic cells 6–10B and S26 presented lower intracellular ROS levels (Figure 6c), indicating that EGFR‐rich H‐EVs induces EGFR up‐regulation and ROS reduction in low metastatic NPC cells and may therefore play a novel non‐redundant promoting role in NPC cells survival and metastasis *in vivo*.

6–10B and S26 ascitic cells were then injected subcutaneously in BALB/c nude mice to generate a subcutaneous transplantation mouse model. H‐EVs (10 μg) were injected into the centre of the resultant xenograft tumours. H&E staining revealed that H‐EVs injection advanced metastasis in the lungs and livers, leading to an increase in the number of tumour nodules, lung weight, and complete loss of pulmonary and hepatic architecture, including vascular structures and germinal centres, of mice bearing 6–10B and S26 xenografts compared with vehicle injection (Figure 6d, Figure 6e and Figure 6f). Moreover, H‐EVs and low metastatic NPC cells co‐transplanted mice displayed significantly shorter survival than vehicle and low metastatic NPC cells co‐transplanted mice (Figure 6g). Collectively, these data, along with the above *in vitro* and *in vivo* results, indicate that highly metastatic NPC cells can secrete EVs that express greater amounts of EGFR. These EGFR‐rich EVs are absorbed by low metastatic NPC cells *in situ* and induce decreased intracellular ROS levels to accelerate the metastasis of poorly metastatic NPC cells (Figure 6h).

## 4 DISCUSSION

NPC is a common head and neck malignancy and the incidence rate has reached 10 per 300,000 (Chan et al., 2013; Lian, Li, & Yu, 2019). Once diagnosed, 74.5% of patients present with lymph node metastases and 19.9% of patients have distant metastases. Approximately 1/3 of patients have a survival period of less than 5 years (Huang et al., 1996; Lee et al., 2009; Wei & Mok, 2007). Metastasis is currently the main obstacle in the clinical management of NPC, and is the dominant cause of death in NPC patients (Li et al., 2012; Ouyang et al., 2012). It is therefore particularly important to explore the relevant molecular mechanisms of NPC metastasis. In this study, we found that EGFR is highly expressed in the tumour tissue of NPC patients with efficient metastases, as well as in highly metastatic NPC cell lines. This high expression is accompanied by decreased intracellular ROS levels (Figure 1). We have also demonstrated that EVs can transfer from highly metastatic NPC cells to poorly metastatic NPC cells for cell communication, and the presence of these EVs can mediate the up‐regulation of EGFR and the down‐regulation of ROS in poorly metastatic NPC cells (Figure 2 and Figure 4). To further explore the role of EVs‐mediated communication for NPC metastasis, we demonstrated that H‐EVs‐mediated EGFR overexpression down‐regulates ROS levels through PI3K/AKT signalling, thus promoting the low metastatic potential NPC cells to the same degree of metastasis as highly metastatic NPC cells (Figure 3 and Figure 5). This suggests that the transfer of H‐EVs not only serves as an educator to remodel the metastatic potential of poorly metastatic NPC cells, but also act as the accelerator for NPC metastasis. More importantly, we found that H‐EVs treatment promoted NPC progression and shortened the overall survival of mice xenografted with NPC cells (Figure 6g), indicating that H‐EVs accelerates the deterioration of NPC. Further exploration of the specific markers of EVs derived from highly metastatic NPC cells may therefore be of clinical significance for the diagnosis and treatment of NPC.

EGFR is the cell surface tyrosine kinase receptor, and activated EGFR recruits and phosphorylates cytoplasmic signalling molecules, thus initiating downstream signalling cascades including JAK/STATs, MAPK/ERK, and PI3K/AKT, to promote tumour cell proliferation, invasion and distant metastasis (Hynes & Lane, 2005; Wang et al., 2011a; Wang, Zhu, Zhang, & Zhang, 2011). However, despite the intersection of signalling activation in different tumours, there may be a preference for EGFR‐activated signalling transduction for the metastasis of tumour cells. EGFR‐activated STAT3, for example, is critical for lung cancer metastasis (Soon, Leong, Koh, & Tham, 2015). Hyperactivation of the EGFR/MAPK signalling pathway often promotes occurrence and metastasis of hepatocellular carcinoma, and colorectal cancer (Tang et al., 2019; Wang et al., 2016). Activation of the EGFR/PI3K/AKT axis is related to the metastasis of lung adenocarcinoma, intrahepatic cholangiocarcinoma, and hepatocellular carcinoma (Hu, Chen, Lou, Zhang, & Yang, 2020; Tiemin et al., 2020; Zhangyuan et al., 2020). In the present study, we found that EGFR‐rich EVs could induce the up‐regulation of EGFR in NPC cells, and then down‐regulate the intracellular ROS levels through the PI3K/AKT pathway to promote the metastasis of NPC cells (Figure 5). This implies that EGFR may be more likely to mediate the metastasis of NPC cells by activating the PI3K/AKT pathway specifically.

Notably, NPC is widely associated with Epstein‐Barr virus (EBV) infection, which is a major driver of NPC genesis and progression (Lv et al., 2019; Zheng et al., 2012). EBV‐encoded LMP1 upregulates EGFR in epithelial cells and increases the release of EGFR into EVs, leading to the development of epithelial malignancies such as NPC (Kung & Raab‐Traub, 2008; Meckes et al., 2010; Miller, Earp, & Raab‐Traub, 1995). Both LMP1 and EGFR can educate the tumour microenvironment and promote the metastatic potential of NPC (Chen et al., 2020; Li et al., 2020b; Sun, Wang, Shi, Zhu, & Wang, 2020). Although all cell lines in our study were EBV negative, further clinical studies are warranted to determine whether EGFR up‐regulation and enrichment of EGFR in EVs are modulated by EBV status and LMP1 levels in NPC patients with distant metastases. In addition, the membrane proteins carried by EVs often determine organ‐specific tumour metastasis (Ji et al., 2020). EV‐delivered EGFR educates the liver microenvironment and promotes liver metastasis of gastric cancer (Zhang et al., 2017). In line with this, we found that EGFR‐rich EVs enhanced liver injury and shortened the overall survival of NPC transplanted mice (Figure 6d and Figure 6g), highlighting that EGFR‐rich EVs play an important role in determining liver‐specific tumour metastasis. Specifically, EVs EGFR, as a special membrane surface marker, has a promoting effect in tumour progression and metastasis, and targeting EGFR may be therefore a promising strategy for improving the therapeutic outcome and prognosis of cancer patients.

Current research shows that ROS play a promoting role in carcinogenesis by boosting proliferation, invasiveness, and metastasis. Alternatively, they can also play anticarcinogenic roles by inducing cell cycle arrest, apoptosis, autophagy and necrosis, and by inhibiting distant metastases (Cairns, Harris, & Mak, 2011; Chen, McMillan‐Ward, Kong, Israels, & Gibson, 2008; Circu & Aw, 2010; Li et al., 2017; Piskounova et al., 2015). More precisely, ROS levels below a certain limit elicit cancer cell growth and metastasis, while an excess of ROS induces cancer cell apoptosis (Bodega, Alique, Puebla, Carracedo, & Ramirez, 2019; Liou & Storz, 2010; Schieber & Chandel, 2014; Sonowal et al., 2017). However, due to the lack of quantitative studies on the effect of differential ROS levels in patients, determining whether there is an anticancer or cancer‐promoting relationship between intracellular ROS levels and tumour metastasis has been controversial, thus limiting the application of ROS as the target in tumour treatment. In the current study, we demonstrated that the ROS levels in NPC patients with efficient distant metastases were significantly lower than that in patients without distant metastasis. This was further confirmed in NPC cell lines (Figure 1), indicating that ROS inhibits the distant metastasis of NPC cells. Further quantitative determination of the selected boundary threshold of ROS for antimetastatic potential, as well as elucidation of the molecular mechanism of ROS‐mediated NPC metastasis inhibition, may be helpful to promote the application of ROS‐targeted chemotherapeutic drugs in NPC patients.

In summary, our study is the first to reveal that EGFR‐rich EVs derived from highly metastatic NPC cells induce the up‐regulation of EGFR and reduce intracellular ROS levels through the EGFR/PI3K/AKT axis to promote the metastasis of poorly metastatic NPC cells, aggravating the progression of NPC. These findings not only unveil the regulatory role of EVs in the NPC tumour microenvironment, but also further elucidate a complex communication mechanism of NPC metastasis mediated by EVs. As EGFR‐rich EVs are mainly derived from tumour cells for effective metastasis, our finding should also have broad implications for the tumour biology field and may advance the detection and treatment of multiple human tumour metastases.

## CONFLICT OF INTEREST

The authors report no conflict of interest.

## AUTHORS’ CONTRIBUTIONS

Fei Li planned and carried out experiments, analysed data, and prepared the manuscript. Xin Zhao and Rui Sun carried out some experiments, analysed data, and prepared the manuscript. Jinxin Ou, Junyu Huang, Nanyan Yang, Ting Xu, Jingyao Li, Xiner He, Chaoyi Li, and Mo Yang performed the research and analysed data. Qing Zhang (PI) supervised the research, planned experiments, analysed data, and prepared the manuscript. All authors read and approved the final manuscript.
